# Novel Fabrication and Characterization of a Bespoke Ultralow Loading Platinum Nanocluster on Carbon Black Catalyst

**DOI:** 10.1021/acs.jpcc.4c08590

**Published:** 2025-04-01

**Authors:** Richard O.D. Clark, Eman Alharbi, Gazi N. Aliev, Wolfgang Theis, Emerson C. Kohlrausch, Graham Rance, Jesum Alves Fernandes, Neil V. Rees

**Affiliations:** † School of Chemical Engineering, University of Birmingham, Birmingham B15 2TT, United Kingdom; ‡ School of Physics and Astronomy, University of Birmingham, Birmingham B15 2TT, United Kingdom; § School of Chemistry, University Park, University of Nottingham, Nottingham NG7 2RD, United Kingdom; ∥ Department of Physics, College of Science, Qassim University, Buraydah 52571, Saudi Arabia

## Abstract

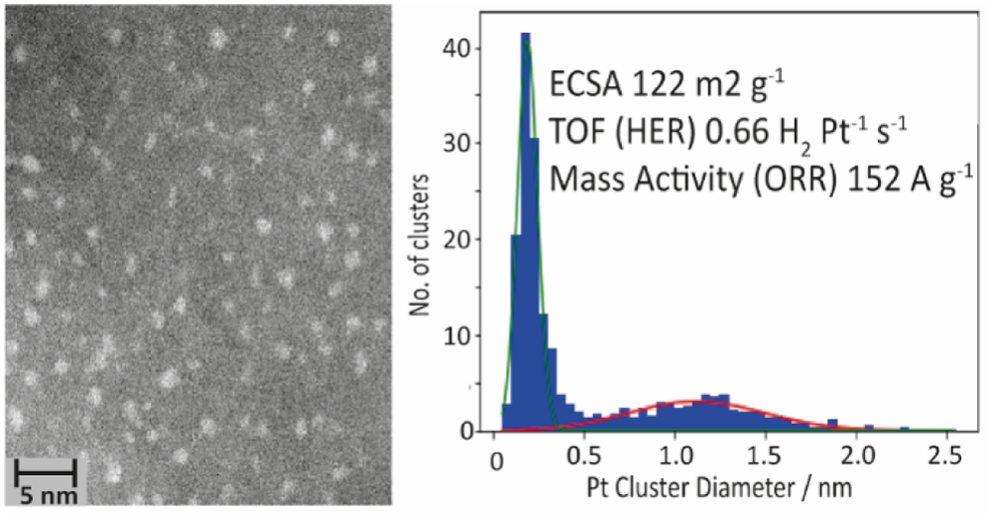

Magnetron sputtering offers a single-step, flexible, and environmentally friendly fabrication route to catalyst production, avoiding the requirement for complex syntheses or toxic chemicals normally required for more traditional wet chemical techniques. Using this facile method, a nanocluster platinum-on-carbon black catalyst is fabricated, rigorously characterized physically and electrochemically, and compared to a well-understood commercial catalyst (TKK). Scanning transmission electron microscopy (STEM) imaging reveals a mean cluster size of 1.1 ± 0.4 nm, half the commercial equivalent, with an associated electrochemically active surface area (ECSA) of 122.2 ± 9.6 m^2^ g^–1^, 40% higher than the commercial comparison. Catalytic performance is measured using the hydrogen evolution reaction (HER) and oxygen reduction reaction (ORR); results indicate a turnover frequency (TOF) 33 times higher than the commercial analogue in the HER and distinct kinetic differences between samples in the ORR. Rotating ring disc electrode voltammetry (RRDE) is utilized to study the mechanism further, and a discussion of activity vs size of particle is presented.

## Introduction

One of the key factors in combating climate change and enhancing long-term energy security is improving the cost-competitiveness of green hydrogen energy alternatives, in particular, polymer-exchange membrane fuel cells (PEMFCs). Central to this is reducing the mass (i.e., cost) of the platinum used in fuel cells; the production of 20 million 50 kW fuel cell vehicles could require around 250 tonnes of platinum,^1^ more than the annual world production of platinum in any year during the period 2010–2021.^2^

One method to reduce the PEMFC/PEM electrolyzer unit cost, as well as aid in the upscaling of production, is to reduce the amount of platinum group metal (PGM) required per unit to generate the required power density. To date, this has broadly been achieved by synthesizing metal particle-on-support catalysts and reducing the amount of “wasted” metal associated with bulk material by increasing the metal surface area-to-volume ratio. Platinum-on-carbon black support catalysts are widely used commercially; such catalysts are commonly fabricated using wet impregnation methods^3^ and contain platinum nanoparticles that are generally ≥ 2 nm in diameter. Platinum has unrivaled activity and stability as a catalyst with respect to the hydrogen evolution/oxidation reaction^4^ and offers exceptional oxygen reduction reaction activity^5^ in acid. The carbon black support is relatively inexpensive and is produced on a multitonne scale every year.^6^ It also has good conductivity and is amorphous, which helps to stabilize the nanoparticles as well as offer a very high surface area.^7^

More recently, significant research activity has been directed toward nanocluster-on-support catalysts, aiming to reduce the size of metal nanoparticles even further, pushing toward the subnanometer domain^8−12^ and the extreme case of single-metal-atom catalysis.^12−16^ Such a strategy maximizes the surface area-to-volume ratio of the catalyst and allows differences in the physical and chemical properties of the catalysts to be probed, exploiting any improvements observed, which can be applied to improve catalyst design.

In this study, platinum-on-carbon black catalysts were prepared via magnetron sputtering, a technique whereby the size of the metal particles being deposited onto the carbon surface can be effectively controlled, producing platinum nanoparticles of ca. 1.1 nm. Following thorough characterization and electrochemical testing, the catalyst is shown to have a significantly higher ECSA, comparable HER activity at 3% loading, and kinetic differences for the ORR compared to the TKK commercial catalyst. These results allow for a better understanding of how catalysts with smaller nanoparticles can be employed for the production and use of green hydrogen in energy applications, reducing the unit cost in the process.

## Experimental Section

### Materials and Methods

The platinum nanoclusters were deposited using bespoke magnetron sputtering with 2.4 wt % of platinum loading. The magnetron sputtering process method is described in detail in the work done by Kohlrausch et al.^17^ Briefly, XC-72R carbon black was placed in an optimized stirring sample holder,^17^ into the sputtering chamber, and placed under vacuum. Argon gas was then introduced, and power was applied (370 V and 200 mA) for 30 min, resulting in highly energetic ions bombarding the platinum target. This led to displaced platinum atoms descending onto the carbon support and bonding with the surface. The carbon black was stirred throughout this process.

For the commercial catalyst comparison TKK with 46.2 wt % platinum/high surface area carbon (HSC) (TEC10E50E, Tanaka Kikinzoku Kogyo K.K.) was chosen. For convenience, the catalysts are referred to as “Pt/C” for the ultralow loading catalyst and “TKK” for the commercial high loading catalyst.

The weight loading for catalyst Pt/C was determined via inductively coupled plasma-optical emission spectroscopy (ICP-OES) (PerkinElmer Optima 8000.) A weighed sample of the catalyst was fully digested in excess aqua regia, and the composition was found to be 2.4 wt % platinum on carbon. The TKK catalyst was also tested using this method and confirmed to be 43.4 wt % platinum on carbon, in good agreement with the quoted value.

The sputtered platinum clusters and single metal atoms were imaged using an aberration-corrected scanning transmission electron microscope (AC-STEM) JEOL-2100F operated in both dark-field and bright-field imaging at 200 kV. The microscope was equipped with a Cs probe spherical aberration corrector (CEOS). The probe convergence semiangle was 19 mrad, and the collection angle ranges of the high-angle annular dark-field (HA-ADF) and annular dark-field (ADF) detectors were set from 72 to 164 mrad (camera length 10 cm) and from 36 to 82 mrad (camera length 20 cm), respectively. STEM images were captured with a probe current of 10 pA and a pixel dwell time of 12–38 μs with a 1024 × 1024 or 512 × 512 pixel scanning area.

Powder X-ray diffraction (PXRD) patterns were obtained using a PANalytical Empyrean diffractometer equipped with a 1° antiscatter slit, and the samples as prepared (catalyst) or from stock (Vulcan) were loaded onto low background Si wafer holders. 2θ values between 5 and 130° were measured with Cu K_α_ radiation (λ = 1.5406 Å) at a scan rate of 4° min^–1^.

The following chemicals were purchased commercially and used without further purification: oxygen (100%, BOC plc), carbon monoxide (99.97%, BOC plc), perchloric acid (70%, VWR Chemicals), 2-propanol (99.9%, Fisher Chemical), ethanol (99%+, Fisher Chemical), sodium perchlorate (≥98.0%, Sigma-Aldrich), Nafion D1021 (Fuel Cell Store), nitric acid (70%, Fisher Scientific), and hydrochloric acid (37%, Honeywell). XC-72R Carbon black (Fuel Cell Store) was used as the support for Pt/C.

All inks were prepared according to literature^18^ to ensure the optimal balance between maximum utilization of platinum particles and mass transport limitations through the catalyst layer. The ink recipes are supplied in the Supporting Information. Further, Kocha et al.^19^ noted that although Nafion does hinder catalyst performance when compared with Nafion-free thin-film layers, Nafion:catalyst ratios of between 0.25 and 1 do not significantly change the catalyst response in rotating disc electrode (RDE) experiments. Therefore, all inks tested consisted of 33 wt % Nafion loading^18^ suspended in solutions containing a combination of ultrapure water (resistivity ≥ 18.2 MΩ cm, Milli-Q, Millipore), 2-propanol, and ethanol. Inks were then sonicated for 60 min (GT Sonic-D6 Ultrasonic Cleaner) and then kept in sealed containers at 278 K between uses. Prior to each use, the inks were sonicated again for a minimum of 30 min to ensure a full dispersion of catalyst material within the ink.

All working electrodes were thoroughly polished with alumina slurries of 1, 0.3, and 0.05 μm sequentially on microcloth pads (Buehler Inc.) Then, 5 μL aliquots of catalyst ink were dropcast onto the electrode face rotating at 700 rpm, as per the method used by Garsany et al.^20^ Due to the large difference in platinum loadings, electrode loadings could not be accurately standardized by mass of platinum on the surface. Instead, catalyst loadings were chosen to ensure a comparable volume of catalyst, ensuring a sufficiently thin, even coverage of the electrode surface so as not to introduce mass transfer resistance. This was then left for a minimum of 15 min, rotating under a heat lamp to ensure the layer dried.

Unless otherwise stated, all electrochemical experiments were thoroughly degassed with nitrogen (oxygen free, BOC plc) with a nitrogen atmosphere maintained throughout and were performed in a standard, three-electrode electrochemical cell with a water jacket thermostated to 298 K. The reference electrode was a HydroFlex reversible hydrogen electrode (Gaskatel GmbH), and the counter electrode was a graphite rod (Goodfellow Cambridge Ltd.). The working electrodes used were glassy carbon rotating disc electrodes (GC-RDE) (Pine Research, 5.0 mm diameter) unless otherwise stated. Cyclic voltammetry (CV) and linear sweep voltammetry (LSV) were performed with an Ivium Technologies Compactstat.e instrument controlled by a computer running IviumSoft software.

The electrochemically active surface area (ECSA) was determined via CO stripping voltammetry. All scans were performed in 0.1 M HClO_4_ electrolyte after cleaning/break-in cycles. The CO saturation and subsequent stripping scan followed the method of Binninger et al.^21^ First, the electrolyte was purged with CO for 10 min with the catalyst-coated electrode submerged and held at 0.1 V vs RHE, before bubbling with N_2_ for 30 min to remove any trace of CO in solution while continuing to hold the electrode at 0.1 V vs RHE, leaving only the adsorbed CO on the Pt surface. The adsorbed layer of CO was then stripped off the platinum by performing cyclic voltammetry, scanning from a potential of 0.1–1.2 V and then returning to 0.1 V (all vs RHE). Following baseline correction, the peak was integrated and the charge was calculated.^22^

Hydrogen evolution reaction (HER) voltammetry was performed in a quiescent solution of 5 mM perchloric acid and 0.1 M sodium perchlorate.

Oxygen reduction reaction (ORR) voltammetry was performed in 0.1 M perchloric acid, saturated with oxygen. During the LSV scans, the solution was kept under an oxygen atmosphere and resaturated between scans. A glassy carbon (GC) rotating disc electrode was used for the LSV scans at a range of rotation speeds within the laminar flow regime.

Rotating ring disc (RRDE) voltammetry was also performed (Pine Research, 5.5 mm diameter GC disc, platinum ring) using the same experimental procedure for the disc electrode responsible for the ORR, and the ring was held at +1.3 V vs RHE for the duration of the experiment. The ring collection efficiency was 38%.^23^

## Results and Discussion

### Physical Characterization

**Figure 1:**
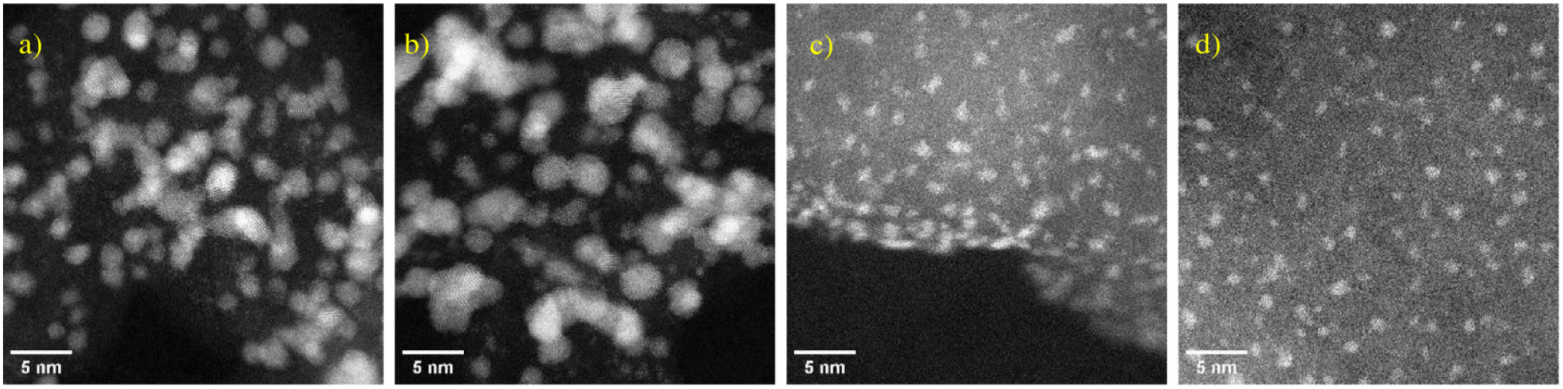
(a,b): Example ADF-STEM images of TKK at 5Mx. Example ADF-STEM images of Pt/C at 5Mx.

**Figure 2:**
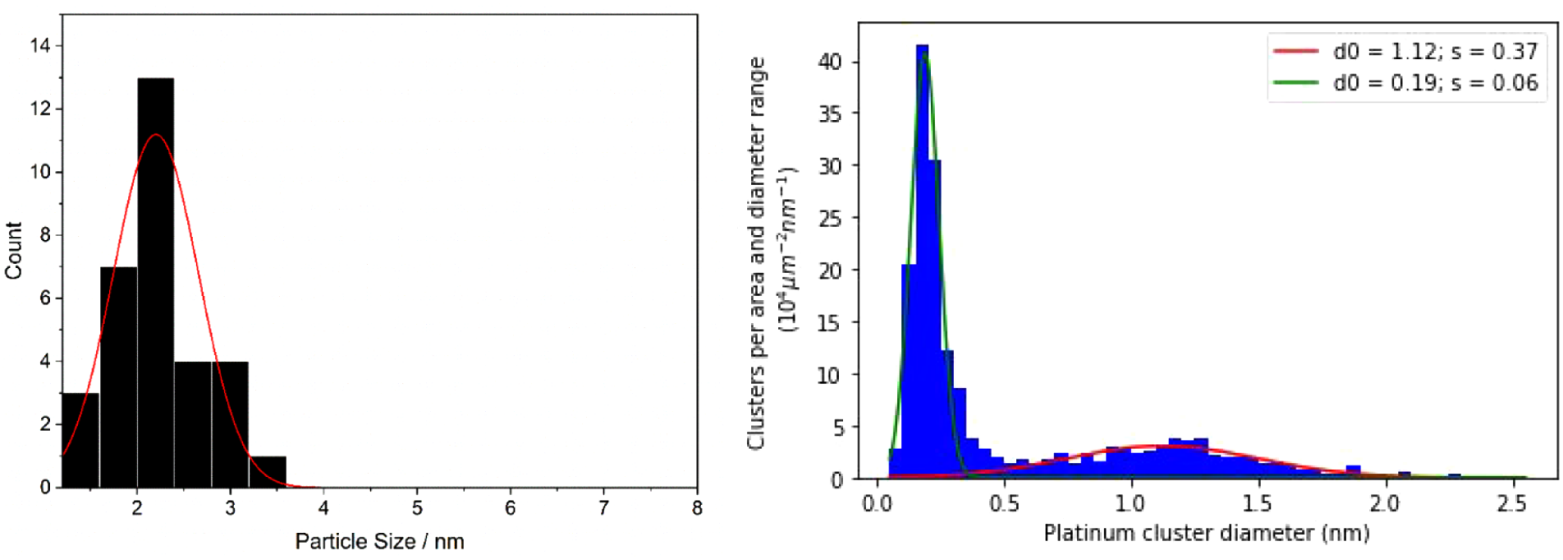
Histograms showing the platinum particle size distribution for TKK (left) and Pt/C (right).

AC-STEM imaging was carried out to evaluate the platinum particles present in the Pt/C sample and to compare them to TKK; examples of images of each are shown in Figure 1. While both samples show a distribution of nanoparticle sizes, it is also worth noting that both have distinct single-metal atoms present across the surface. Given it appeared there were two distinct groupings of platinum particle size, a quantitative size distribution was generated using random sampling and imaging of the catalysts to study their size distribution. Figure 2 shows the resulting histograms for each catalyst, with sample sizes of 32 particles for TKK and 2,415 for Pt/C. Full details of the sampling method are contained in the Supporting Information. Fewer particles were sampled for TKK because only isolated platinum particles were analyzed; due to high loading, many particles overlapped in the imaging.

The mean diameter of the platinum nanoparticle for Pt/C was determined to be 1.1 ± 0.4 nm, but the distribution is bimodal, with a significant number of single-metal atoms also present. TKK shows a mean diameter of nanoparticle size of 2.2 ± 0.5 nm, in good agreement with the 2.3 nm reported by Takahashi and Kocha.^24^ Furthermore, a significant number of single-metal atoms were observed for Pt/C, whereas fewer platinum single atoms were observed for TKK. However, due to the high number of larger particles in TKK, many single atoms were obscured, so a quantitative size analysis could not be determined. Imaging showed that most platinum particles in the Pt/C sample were 2D clusters with no defined crystal structure, in contrast to the larger nanoparticles with a well-defined crystal structure in the TKK sample. This is all in line with the general trend that, on increasing the platinum loading on the support, the particle size will increase in size.

Further physical characterization via powder X-ray diffraction (PXRD) was attempted to support observations in the STEM analysis, specifically the degree of platinum crystallinity observed and platinum particle size. However, a combination of an amorphous carbon support resulting in broad carbon peaks,^25^ low platinum weight loading, and small platinum particles^26^ meant that no distinct platinum peaks could be resolved for Pt/C. This could be due to the absence of well-defined crystal structure or because the platinum loading is below the detection limit for PXRD.^27^ The spectra are given in the Supporting Information.

Further electrochemical characterization was therefore undertaken to probe any differences in activity due to the size and/or crystallinity.

### Electrochemical Characterization

#### Electrochemically Active Surface Area (ECSA)

**Figure 3:**
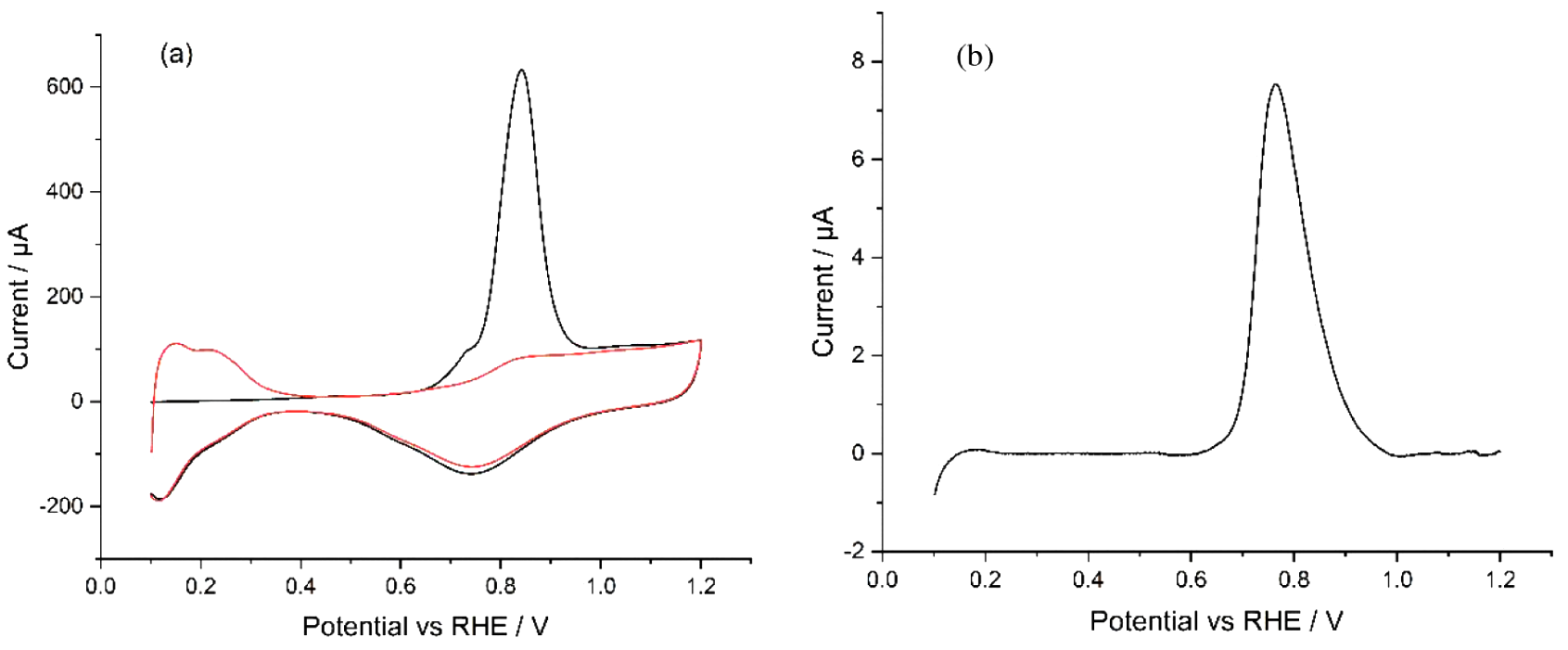
(a) First (black) and second (red) voltammograms following CO saturation of the TKK platinum on the carbon catalyst in nitrogen-saturated solution containing 0.1 M HClO_4_, at a voltage scan rate of 20 mV s^–1^. (b) Typical baseline-corrected CO stripping LSV for our fabricated Pt/C in nitrogen-saturated solution containing 0.1 M HClO_4_, at a voltage scan rate of 10 mV s^–1^.

To find the ECSA, carbon monoxide stripping was utilized for both TKK and Pt/C. Figure 3a illustrates a typical CO stripping response: in the first cycle, the hydrogen desorption is suppressed, indicating full saturation of the surface with CO. Following the stripping peak, the hydrogen adsorption/desorption features reappear in the second cycle.^21^ A typical baseline-corrected stripping peak for Pt/C is shown in Figure 3b, and analysis of several such experiments for both samples yielded ECSA values of (122.2 ± 9.6) m^2^ g^–1^ for Pt/C and (88.0 ± 4.3) m^2^ g^–1^ for TKK.

#### Hydrogen Evolution Reaction (HER)

**Figure 4:**
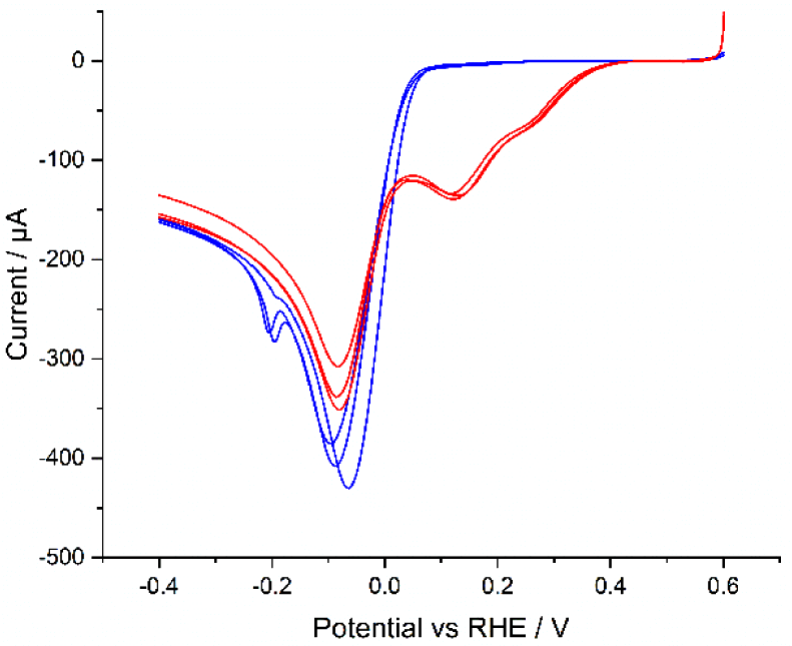
Linear sweep voltammograms for TKK (red) and Pt/C (blue) in a solution of 5 mM HClO_4_ and 0.1 M NaClO_4_ at a voltage scan of 20 mV s^–1^.

The HER was used as a well-known test system with fast kinetics on nanoplatinum to investigate and compare the activity of Pt/C to TKK. Voltammetry was conducted in quiescent solution containing 5 mM HClO_4_ and 0.1 M NaClO_4_, and Figure 4 shows the resulting baseline-corrected linear sweep voltammograms for Pt/C vs TKK. While there is some interference from hydrogen underpotential deposition (H_UPD_), the peak potential is approximately the same for both samples, despite Pt/C having 3% of the platinum loading on the electrode surface (1.2 μg cm^–2^ vs 40 μg cm^–2^).

A comparison of the peak currents (*i*_p_*)* obtained experimentally with that predicted by the Randles–Ševčík equation:^28^
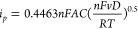
1

(where *T* = 298 K, *v* = 0.02 V s^–1^, *D* = 9.3 × 10^–5^ cm^2^ s^–1^,^29^*n* = 1,^30^*C* = 5 × 10^–6^ mol cm^–3^, *A* = 0.196 cm^2^, *R* = 8.314 J K^–1^ mol^–1^, and *F* = 96,485 C mol^–1^) shows good approximate agreement, with a value of 360 μA. This indicates that the catalyst deposits follow case 4 diffusive behavior,^31,32^ that is, both catalysts have sufficient dispersion of the platinum particles to ensure full utilization of the electrode surface.

The comparison between the two catalysts is shown in Table 1. Pt/C shows an earlier onset (defined here as the potential at which the current passes 50 μA) and half-wave potentials,^31^ and Pt/C has 33 times the TOF of TKK at 0 V. The TOF is calculated by:
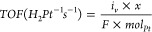
2where *i*_*v*_ is the current at 0 V in amps, *mol*_Pt_ is the moles of platinum on the electrode surface, and *x* is ; the stoichiometric value from the equation:
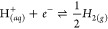
3

**Table 1:** HER Comparison between TKK and Pt/C

	TKK	Pt/C
Peak Potential/mV	–83 ± 2	–83 ± 17
Onset Potential (Taken at 50 μA)/mV	0 ± 4	25 ± 6
Half-Wave Potential/mV	–31 ± 2	–17 ± 11
Turnover Frequency at 0 V/H_2_ Pt^1–^ s^–1^	0.02 ± 0.00	0.66 ± 0.21
Tafel Slope/mV dec^–1^	31 ± 2	32 ± 4

These results can be rationalized by Ward et al.^31^ who showed that peak potential and/or onset potential differences can be misinterpreted as an inherent improvement in catalytic effect when it can be due to changes in the geometry of the surface (including structural or electronic factors). Here, the same peak potential is found, despite the coverages on the electrode, Ψ, being very different:
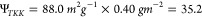
4
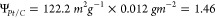
5


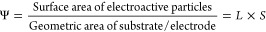
6

where *L* is the platinum loading and *S* is the ECSA.

The standard heterogeneous rate constant, *k*_0_, for the HER is of the order of 10^–2^ cm s^–1^, as per Qin et al.^33^ This is at the limit of reversibility, since Matsuda and Ayabe^34^ defined the reversibility threshold at stationary macroelectrodes at 298 K as
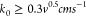
7Hence, the threshold for reversibility in this case (voltage scan rate, *v* = 20 mV s^–1^) would be 4.2 × 10^–2^ cm s^–1^. Therefore, any shift in peak potential due to an improvement in kinetics would not be discernible (within experimental error) at this scan rate through shifts in peak potential.

Hence, the earlier onset and half-wave potentials strongly suggest the observed differences are due to mass transport effects^35^ associated with the catalyst layer and not a kinetic improvement. This may be linked to the nature of the carbon blacks used as supports in the samples, with the XC-72R (used for Pt/C) having a considerably lower porosity than the proprietary “HSC” carbon black used for the TKK catalyst. It is postulated that the improved activity of Pt/C over TKK is linked to the location of the Pt clusters/particles on the nonporous/porous carbon black support particles. The platinum particles in Pt/C are located on the surface of XC-72R due to its lower porosity and associated surface area of 160 m^2^ g^–1^, whereas a significant proportion of the platinum particles in TKK are likely located within nano, meso, and macropores of the highly porous, higher surface area (527 m^2^ g^–1^) HSC carbon.^36^ In turn, the detachment of hydrogen bubbles formed would be expected to occur more easily from the surface-located platinum particles (Pt/C) compared to those from those platinum particles located in pores (TKK), leading to a lower charge transfer resistance for XC-72R versus the HSC carbon due to the bubbles hindering access of the electrolyte to the platinum surface. In situ experimental confirmation of this effect is extremely challenging at the nanoscale, and future work will seek to study this effect.^37,38^

Finally, both catalysts show Tafel slopes ca. 30 mV dec^–1^, indicating both follow a Volmer + Tafel mechanism, where the Tafel step is the rate-determining step (RDS).^39^
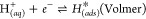
8
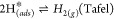
9

Hence, Pt/C can produce comparable HER activity to TKK with only 3% of the platinum loading via more efficient use of the platinum atoms present within the catalyst.

#### Oxygen Reduction Reaction (ORR)

**Figure 5:**
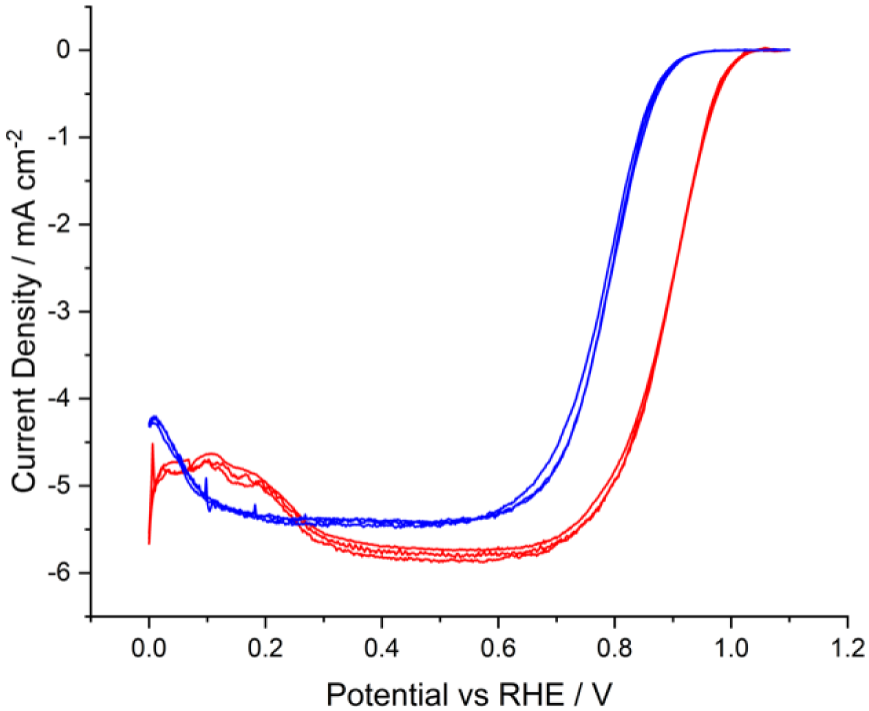
Linear sweep voltammograms of TKK (red) versus Pt/C (blue) in a saturated solution of O_2_ in 0.1 M HClO_4_ recorded at a voltage scan rate of 20 mV s^–1^ and rotation speed of 1600 rpm at 298 K.

To further compare the electrocatalytic properties of TKK to Pt/C, the oxygen reduction reaction was investigated as a key catalytic reaction with slower kinetics, where catalytic differences between Pt/C and TKK may be observable. Both catalysts were drop-casted onto the surface of a 5 mm GC electrode, which was then immersed in an oxygen-saturated solution of 0.1 M HClO_4_, and LSV under rotation was utilized to probe differences between catalysts while controlling the rate of mass transport.^40^ The resulting voltammograms at 1600 rpm are shown in Figure 5.

The results show the TKK onset potential is around 100 mV earlier than Pt/C, leading to a larger current density at 0.9 V. Considering the surface coverage (Ψ) effects^41^ outlined earlier, the irreversibility of the ORR^42^ causes a shift in the onset due to the value of Ψ described by the following equation:^43^
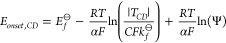
10where α is the transfer coefficient,  is the formal reduction potential,  is the formal electrochemical rate constant for the reduction at the active platinum sites,^44^ Ψ is the surface coverage, as defined in eq 6, C is the concentration, *T* is the temperature, and *E*_*onset,CD*_ is the onset potential as defined via a threshold current density |*T*_*CD*_| (here 500 μA cm^–2^) within the Tafel region of the voltammogram. The value of α was 1.0 ± 0.1 for both TKK and Pt/C, calculated via mass transport-corrected Tafel analysis.^45^ This agrees with the Damjanovic analysis of the ORR mechanism,^46^ discussed further below.

Since this study is comparing Pt/C to TKK, eq 10 can be used to calculate the relative shift in onset potential:
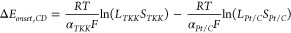
11

The voltammograms in Figure 5 show an experimental difference in onset potentials (Δ*E*_*onset,CD*_) of 102 mV (measured at the threshold current density |*T*_*CD*_| = 500 μA cm^–2^). This is greater than the difference of 77 mV, calculated by eq 11, and the remaining difference of 25 mV can therefore be attributed, via eq 10, to a lower  for Pt/C than TKK. This is consistent with literature reports that the oxygen reduction reaction activity decreases as particle size decreases^47,48^ due to the increasing oxophilicity of smaller particles, which leads to OH_ad_/O_ad_ blocking active sites. The difference here can only be due to kinetics; both carbon black supports are highly conductive,^49,50^ negating electrical resistance as a significant effect. Further, porosity differences affecting mass transport of oxygen to platinum can also be disregarded since the onset region of the voltammogram is also the region dominated by kinetics.^51^ Indeed, an assumption of the Tafel slope calculated and shown in Table 2 is that there is negligible contribution from mass transport.^52^ If there was a contribution, then a difference in Tafel slope would be observed between samples arising from an additional resistive (mass transport) term.

**Table 2:** Key Parameters for the ORR on TKK Vs Pt/C.^a^

	TKK	Pt/C
Effective Number of Electrons, n	3.8	3.6
Specific Activity @ 0.9 V/A m^–2^	3.2 ± 0.3	1.2 ± 0.2
Mass Activity @ 0.9 V/A g^–1^	280 ± 26	152 ± 20
Tafel Slope/mV dec^–1^	60 ± 1	60 ± 0

a All scans used were performed at 1600 rpm, 20 mV s^–1^, sat. O_2_

As further evidence of this, Table 2 shows the specific and mass activities calculated from the normalized data of each catalyst tested. As expected, Pt/C not only shows decreased specific activity, in line with the  analysis above, but also shows a decrease in mass activity. This shows that, on reducing the size of the particles from 2.2 nm (in this case, 1.1 nm), the increase in surface area-to-volume ratio associated with smaller particles does not provide enough extra utilization of platinum mass to overcome this reduction in specific activity.

Figure 5 also indicates that both catalysts approach the limiting current for platinum catalysts reported in the literature of circa. 5.7–6 mA cm^–2^.^53−56^ The approximate theoretical maximum limiting current density for the ORR can be calculated by using the Levich equation:
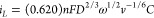
12

where *i*_*L*_ is the Levich current, *n* is the number of moles of electrons transferred, *D* is the diffusion coefficient of the reagent, ω is the angular rotation rate in rad s^–1^, ν is the kinematic viscosity, *C* is the concentration of the reagent, and *F* is the Faraday constant.

Taking *n* = 4, *D* = 1.93 × 10^–5^ cm^2^ s^–1^, ν = 1.01 × 10^–2^ cm^2^ s^–1^, and *C* = 1.26 × 10^–6^ mol cm^–3^,^57^ a value of 6.0 mA cm^–2^ at 1600 rpm is calculated. It is worth noting the difference in current between TKK and Pt/C below 0.3 V, which arises from the presence of desorbing hydrogen ions from the platinum surface, which contributes non-Faradaic current^58^ that offsets the current generated by the ORR. This is distinct in the case of TKK due to the much higher platinum loading, as well as the facets present within the crystalline nanoparticles.^59,60^ In contrast, Pt/C has a much lower loading, which reduces the magnitude of any observable desorption current and an absence of crystal structure, leading to a less well-defined desorption region.

As with the HER, there are a number of factors that contribute to whether the theoretical limiting current is reached for an electrochemical process involving a drop-cast catalyst onto an electrode surface. These factors include: catalyst film/layer homogeneity,^53^ catalyst coverage of the electrode surface,^41^ catalyst loading, anddispersion of active material (here platinum) across the support (here carbon) on the catalyst,^61^ and the Faradaic efficiency of the reaction studied.

The degree of homogeneity of the catalyst layer has a strong effect on the voltametric response. The layer should be sufficiently thin and uniform to prevent any contributions from convergent diffusion due to surface roughness^41^ to ensure that diffusion to the surface is linear and therefore can be analyzed using eq 12. In addition, the catalyst layer should cover the entire working surface of the electrode since it depends on the geometric area of the electrode (not the amount of electroactive material present) (eq 12). Similarly, the active material (platinum) needs to be evenly dispersed across the support (within the layer) to avoid inactive zones that may also compromise the validity of eq 12. Last, in the case of multielectron mechanisms such as the ORR, the exact number of electrons transferred (*n*) can be difficult to ascertain: for example, the ORR is usually considered to operate via a mix of 2e^–^ and 4e^–^ pathways depending on the catalyst and substrate.^62^

**Figure 6:**
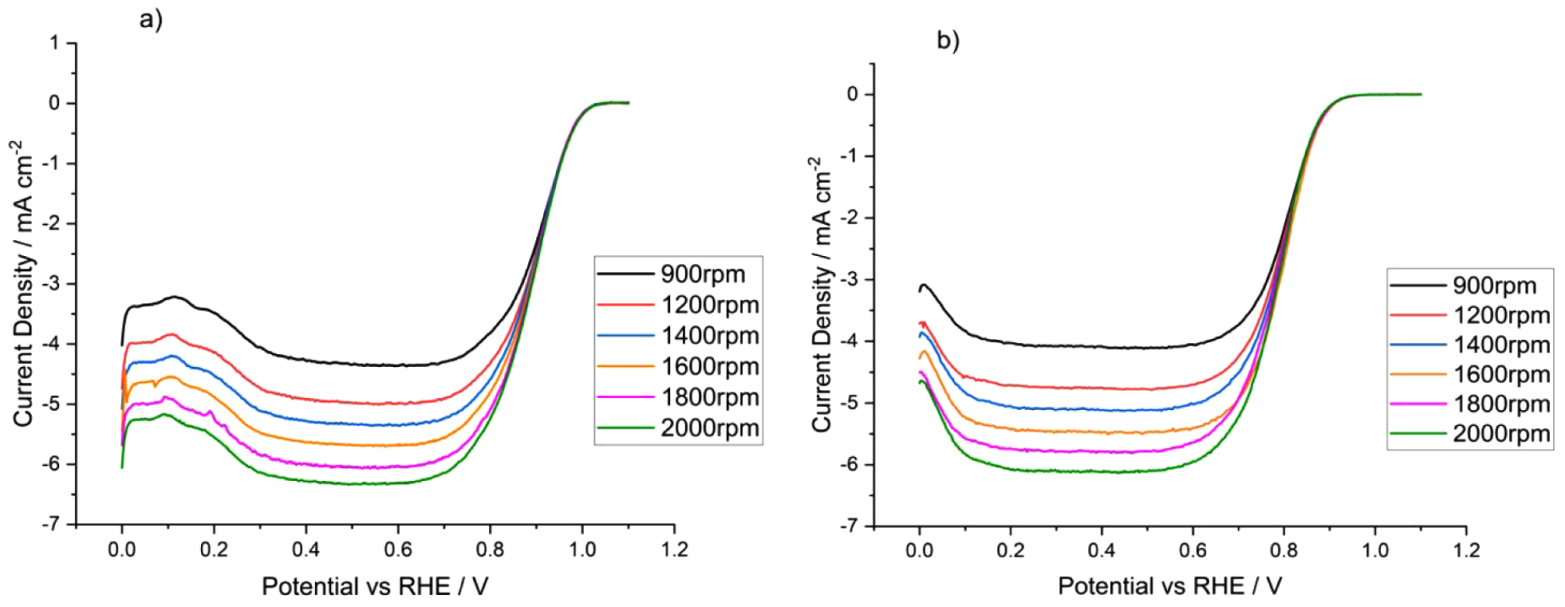
Linear sweep voltammograms at various rotation speeds for (a) TKK and (b) Pt/C in 0.1 M HClO_4_, 20 mV s^–1^, sat. O_2_.

To probe these differences, the activities of the catalystswere recorded as a function of rotation rate; Figure 6 illustrates the resulting voltammograms. The limiting currents were then used to calculate the number of electrons in the mechanism via plotting the Levich spectra (eq 10). The number of electrons for each is shown in Table 2.

Neither catalyst reaches *n* = 4, in part due to the nonideal surface, but the extent to which Faradaic efficiency and/or quality of dispersion of platinum across the carbon black influences the calculated value of *n*, as well as the differences between samples, can be investigated via rotating ring disc electrode voltammetry, in particular why Pt/C has a lower value for *n* than TKK.

**Figure 7:**
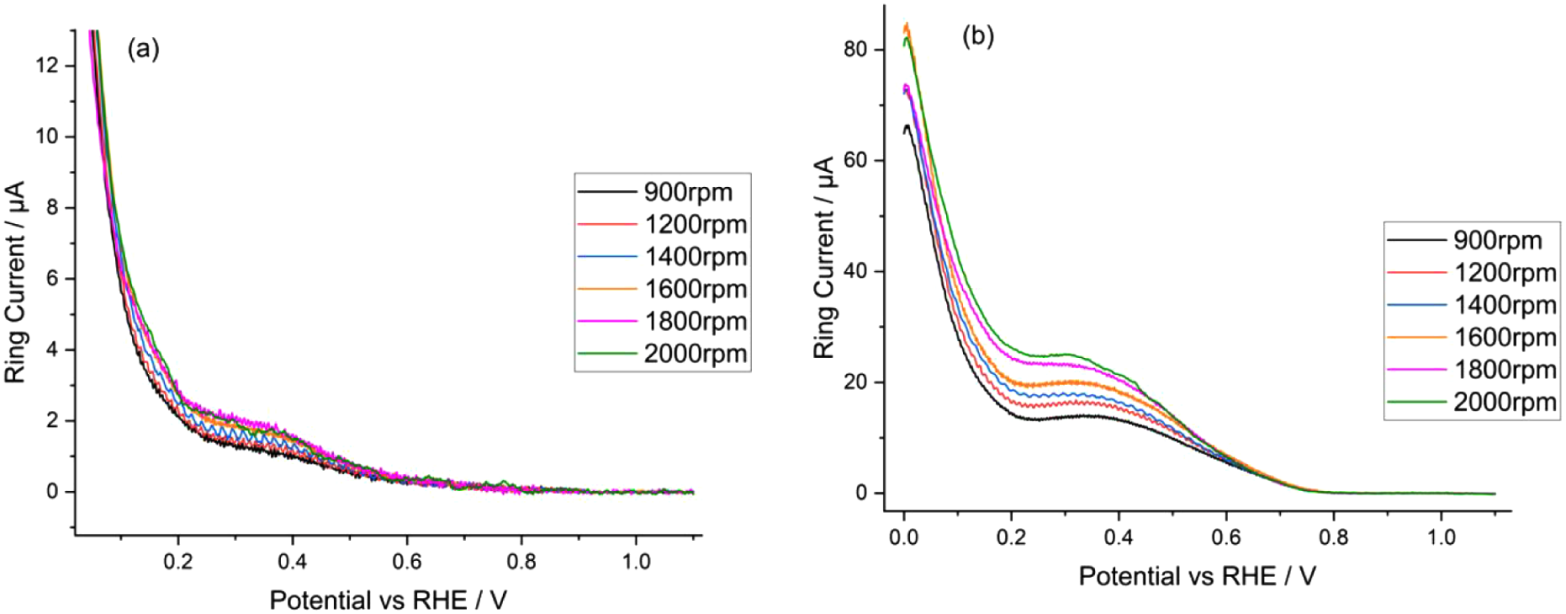
Ring current linear sweep voltammograms at various rotation speeds for (a) TKK and (b) Pt/C in a solution of O_2_-saturated 0.1 M HClO_4_ at a voltage scan rate of 20 mV s^–1^.

Figure 7 shows the ring current linear sweep voltammograms for each of the catalysts tested as a function of the rotation rate. It is clear that Pt/C produces an order of magnitude more ring current, and hence hydrogen peroxide, when compared to TKK (ca. 20 μA vs 2 μA at 1600 rpm), which indicates a preference for the two-electron pathway:
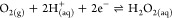
13over the direct, four-electron pathway:
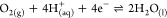
14Thus, the number of electrons calculated in the Levich analysis above is lower for Pt/C vs TKK.

To confirm whether the decrease in Levich calculated number of electrons from 3.8 to 3.6 is solely due to the 2e^–^/4e^–^ pathways, and not, for example, also due to the dispersion of platinum across the carbon black, the number of electrons transferred was calculated via a ratio of the disc to ring current:^63^
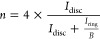
15where *I*_disc_ and *I*_ring_ are the disc and ring currents, respectively, and *B* is the collection efficiency of the ring (38%).

Using the RRDE voltammetry, eq 15 indicates that in the mass transport limited region, there is a difference in *n* values of 0.2 due to the relative disc and ring currents, i.e., the lower *n* values for Pt/C is purely due to its lower selectivity toward the 4-electron pathway than TKK. It can therefore be concluded that there is no loss in the calculated value of *n* in the Levich analysis from poor dispersion of platinum on the carbon black.

The preference for hydrogen peroxide production also suggests further evidence of the difference in the structure between the two catalysts. Taylor et al.^61^ found that hydrogen peroxide production increases as platinum loading decreases due to the increasing interparticle distance, meaning that there are fewer active sites in direct proximity to the reaction site that are able to readsorb any hydrogen peroxide formed to allow it to react further to produce water. In addition, platinum single-metal atoms are more selective to the two-electron reduction to hydrogen peroxide than clusters or nanoparticles.^64,65^ This observation further supports the STEM analysis of the Pt/C sample having a higher density of single-metal atoms than TKK and also that the particles are likely to be further apart from one another.

Finally, both catalysts show Tafel slopes of approximately 60 mV dec^–1^,^66,67^ indicating the widely accepted Damjanovic mechanism^46,68^ of:
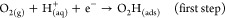
16
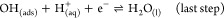
17

As summarized by Antoine et al.,^69^ the mechanism is reaction order 3/2 with respect to H^+^ activity, not 1, and surface concentrations of adsorbed intermediates vary linearly with pH and potential per the Temkin isotherm.

## Conclusions

A combination of physical, electrochemical, and related studies in the literature have been utilized to critically analyze magnetron-sputtered platinum nanoclusters on Vulcan carbon black catalysts. It has been shown that this method can fabricate catalysts with stable nanoclusters (particle size 1.1 ± 0.4 nm) significantly smaller than a commercial equivalent, including a relatively high density of single-metal atoms, to be utilized in catalytic applications. This catalyst has yielded a high ECSA of 122.2 ± 9.6 m^2^ g^–1^, superior HER activity via increased platinum utilization (TOF of 0.66 ± 0.21 H_2_ Pt^1–^ s^–1^ vs 0.02 ± 0.00 H_2_ Pt^1–^ s^–1^ for TKK), and its smaller sized particles are sufficiently stable to generate a quantitative difference in ORR kinetics.

This demonstration of producing well-defined and stable platinum clusters with sizes close to the subnanometer domain, on a multigram scale, will enable future work to utilize this fabrication technique to explore the large-scale production of subnanometer metal particle-on-support catalysts and understand the key factors in optimizing catalyst activity.

## Supporting Information Available

quantitative size distribution analysis, XRD results, catalyst layer imaging, rotating ring-disk voltammetry, and accompanying Levich analysis (PDF)
