# Supplementary material for: Novel Fabrication and Characterization of a Bespoke Ultralow Loading Platinum Nanocluster on Carbon Black Catalyst
Source: J Phys Chem C Nanomater Interfaces. 2025 Apr 1;129(14):6739–48. doi: 10.1021/acs.jpcc.4c08590 (PMC11998067; doi:10.1021/acs.jpcc.4c08590)
Supplement: Supplementary file 1 — jp4c08590_si_001.pdf [file jp4c08590_si_001.pdf]

Novel Fabrication and Characterisation of a Bespoke Ultra-Low Loading Platinum Nanocluster on Carbon Black Catalyst

Richard O.D. Clark,[a] Eman Alharbi,[b,d] Gazi N. Aliev,[b] Wolfgang Theis,[b] Emerson C. Kohlrausch,[c] Graham Rance,[c] Jesum Alves Fernandes,[c] and Neil V. Rees[a]*

[a] School of Chemical Engineering, University of Birmingham, Birmingham, B15 2TT, United Kingdom.

[b] School of Physics and Astronomy, University of Birmingham, Birmingham, B15 2TT, United Kingdom.

[c] School of Chemistry, University Park, University of Nottingham, Nottingham, NG7 2RD, United Kingdom.

[d] Department of Physics, College of Science, Qassim University, Buraydah 52571, Saudi Arabia.

Supporting Information

S1: Worldwide Platinum Production[1]

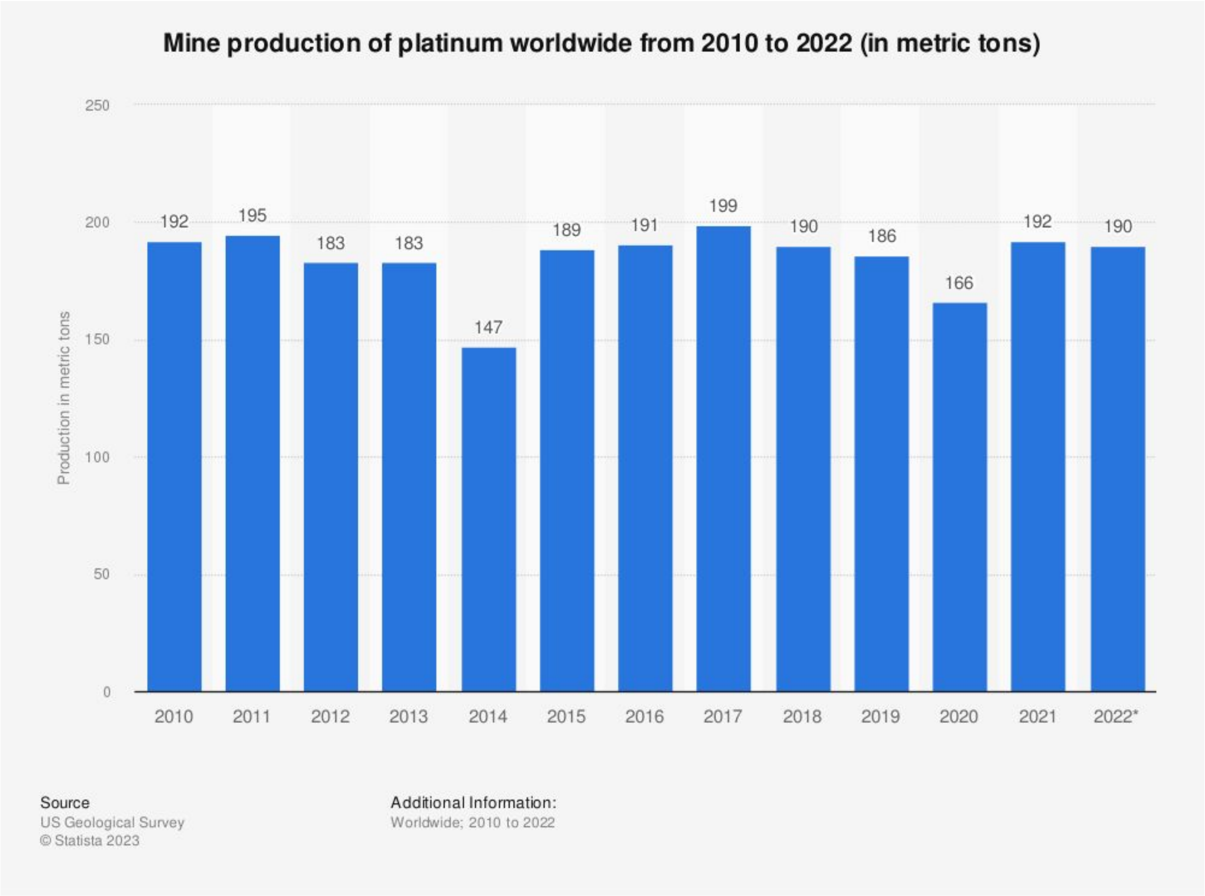

S2: Quantitative Size Distribution Analysis Via ADF-STEM Imaging

As illustrated in **Figure S2**, the TKK catalyst shows large and joined up platinum particles when imaged using ADF-STEM. As a result, it was difficult to isolate single clusters from these larger, joined particles. ImageJ was therefore used to pick up the clearest particles manually, measure their projected areas, and then calculate the mean diameter; the size distribution of the TKK catalyst platinum particles is shown alongside the image used in **Figure S2**. Most particles lie within the range of 1.5 to 3.3 nm in diameter. The mean diameter is 2.2 nm and the standard deviation of the measured diameters is 0.5 nm.

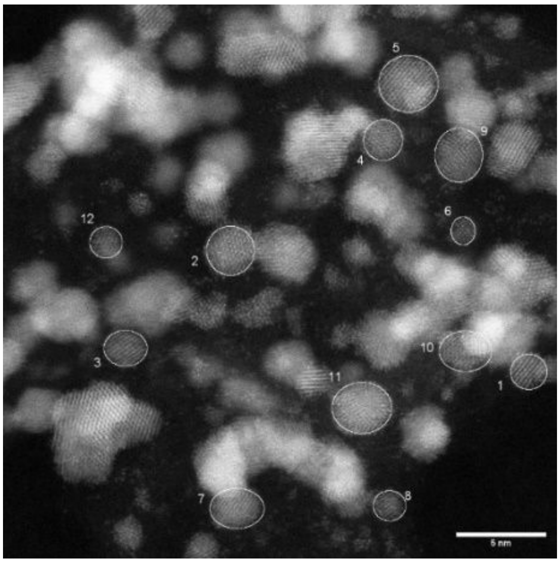

Figure S2 ADF-STEM image of TKK at high magnification (5Mx).

Turning to Pt/C, 6 different sized carbon black support flakes are investigated. Two Gaussian distributions were fit to the data. The Gaussian at 1.12 nm with a standard deviation of 0.37 nm represents clusters in a 1.0 nm to 1.4 nm diameter range. The Gaussian at 0.2 nm corresponds to single atoms. This analysis was conducted on different carbon black support flakes randomly chosen within a specific grid region. The same analysis was performed on each. The nanoparticle diameters in these supports are 1, 1.1, 1.2, and 1.4 nm. These values were then weighted according to the relative area provided by each support. Considering the area each support flake contributes relative to the others, a histogram was generated to represent clusters per square micrometre (clusters/μm^2), as shown in S2B.

S3: SEM Imaging of Catalyst Layer on Glassy Carbon Surface.

Scanning electron microscopy (SEM) images were recorded on a Hitachi TM3030 tabletop SEM. Samples were drop-cast onto 5mm GC electrodes and left to air dry, before being mounted onto the sample holder for analysis. Back scattered electron (BSE) analysis was primarily used to highlight areas of higher Pt density, and EDX was used for elemental dispersion.

SEM was used for imaging of the catalyst powder to observe the dispersion of platinum across the carbon support, whether aggregates large enough to detect with SEM imaging were present and hence gauge the homogeneity of the dispersion. Figure S4A shows the resulting back-scattered electron (BSE) image of the TKK layer; Figure S4B shows the same for Pt/C. On first inspection both samples appear to contain aggregates: elemental mapping at 1500 times magnification suggests that Pt/C has occasional platinum aggregates of up to 15 µm present, showing that the platinum has not been evenly distributed.

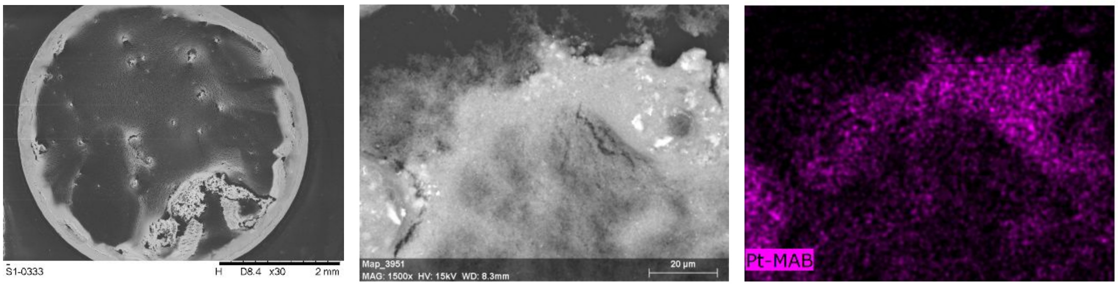

Figure S3A: SEM Imaging of TKK layer on glassy carbon electrode at x30 magnification (left) and at x1500 in a brighter region (middle) with associated elemental mapping (right).

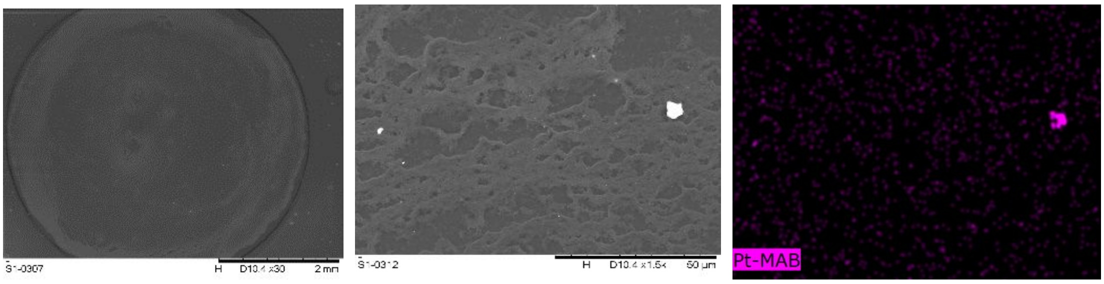

Figure S3B: SEM Imaging of Pt/C layer on glassy carbon electrode at x30 magnification (right) and at x1500 in a region with a bright speck (middle) with associated elemental mapping (right.)

S4: Levich plots of TKK (left) and Pt/C (right) with calculated number of electrons.

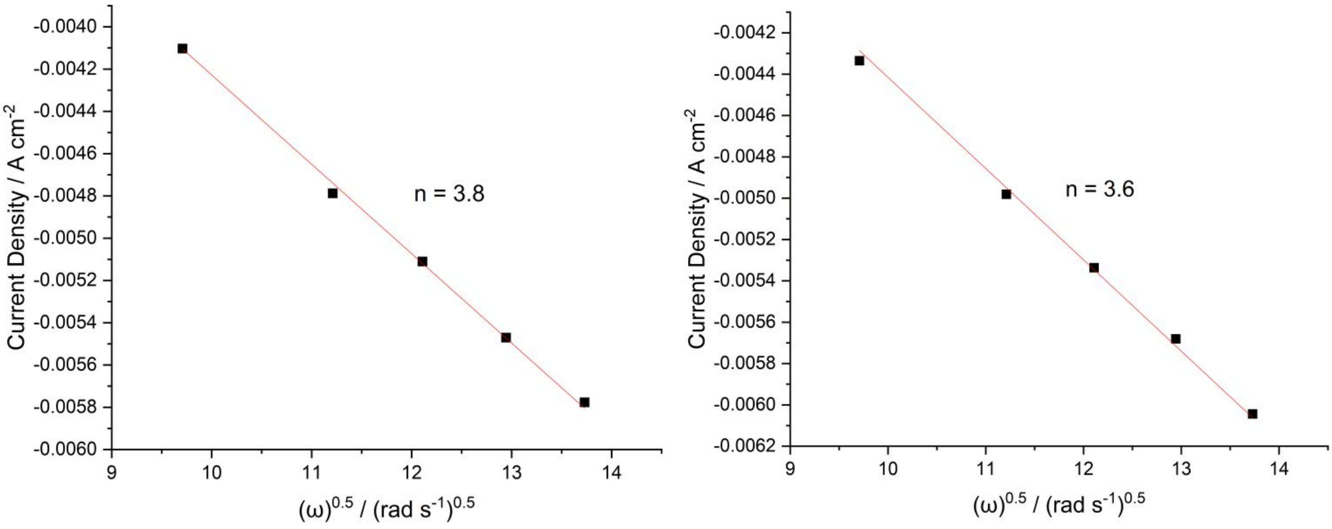

S5: Rotating ring disc electrode voltammetry: disc current vs rotation rate

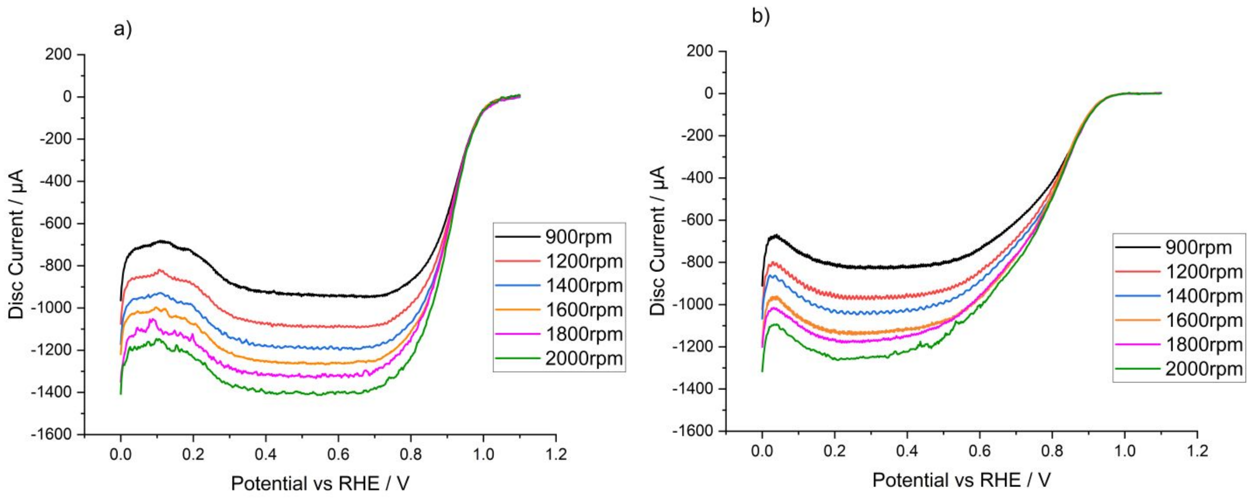

Figure S5: Disc current vs potential in 0.1 M $HClO_4$, 20 mV s^{-1}, sat. O_2 for TKK (a) and Pt/C (b)

Figure S5 provides the associated disc currents that accompany the ring currents shown in **Figure 6** in the main paper.

S6: Ink recipes for each catalyst tested

		TKK	Pt/C
Weight of Catalyst	g	0.0034	0.0020
Nafion	mL	0.0160	0.0094
Propan-2-ol	mL	0.2000	0.2480
Ethanol	mL	0	0.4950
Deionised Water	mL	0.7840	0.2480

Both recipes were chosen to ensure an even and thin layer of catalyst on the electrode surface. The composition of propan-2-ol, ethanol and deionised water are based on the work of others for TKK[2] and Vulcan XC-72R[3] and differ due to the different physical properties of each carbon black. Nafion composition was chosen based on the work of Passalaqua et al[4]. to ensure that ink recipes would be consistent from half cell testing through to single cell testing, as necessary.

S7: Powder x-ray diffraction spectra collected for Vulcan XC-72R support only, and Pt/C

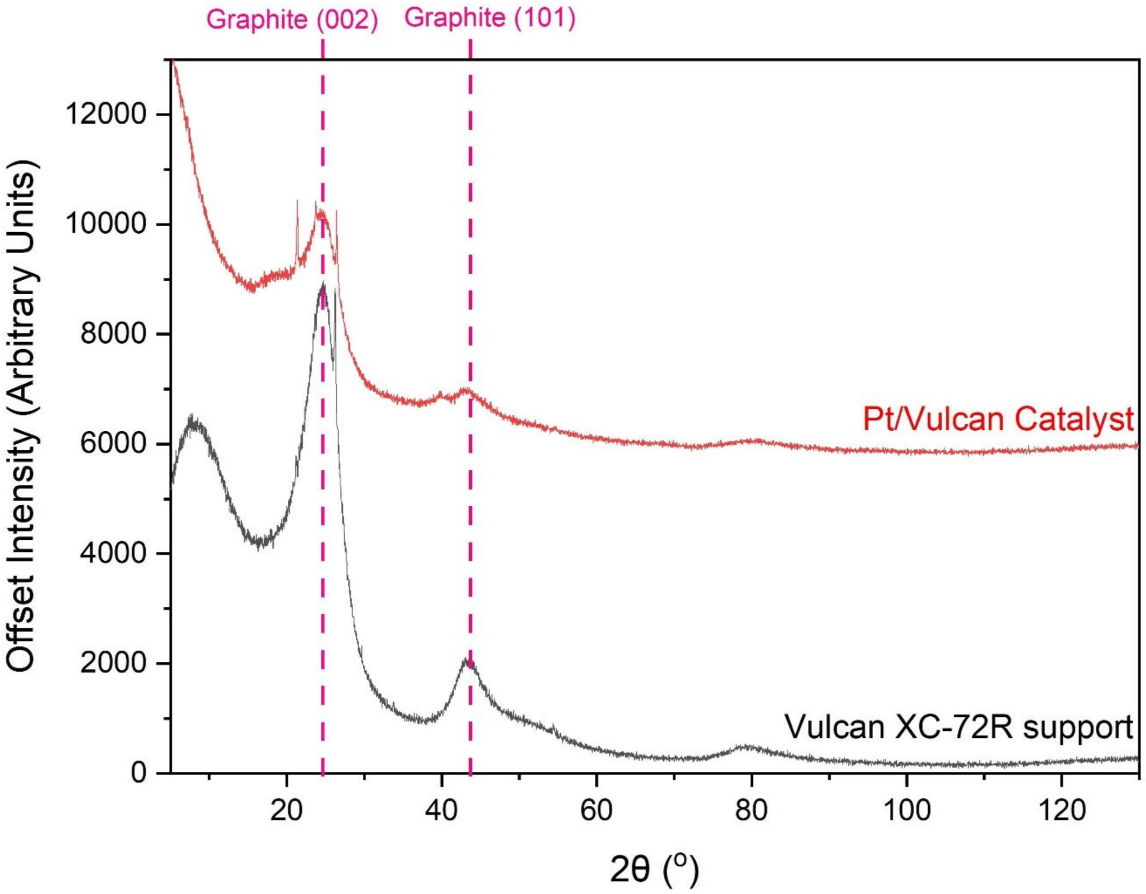

References

1 U. Geological Survey, *Mineral Commodity Summaries 2022*, .

2 S. S. Kocha, K. Shinozaki, J. W. Zack, D. J. Myers, N. N. Kariuki, T. Nowicki, V. Stamenkovic, Y. Kang, D. Li and D. Papageorgopoulos, *Electrocatalysis*, 2017, **8**, 366–374.

3 J. Soto-Pérez, L. E. Betancourt, P. Trinidad, E. Larios, A. Rojas-Pérez, G. Quintana, K. Sasaki, C. J. Pollock, L. M. Debefve and C. R. Cabrera, *ACS Omega*, 2021, **6**, 17203–17216.

4 E. Passalacqua, F. Lufrano, G. Squadrito, A. Patti and L. Giorgi, *Nafion content in the catalyst layer of polymer electrolyte fuel cells: effects on structure and performance*, 2001, vol. 46.
